# The Regenerative Effect of Bone Marrow-Derived Stem Cells in Spermatogenesis of Infertile Hamster

**Published:** 2017-01

**Authors:** Akbar Vahdati, Alireza Fathi, Mehrdokht Hajihoseini, Ghaem Aliborzi, Ebrahim Hosseini

**Affiliations:** 1 Department of Biology, Fars Science and Research Branch, Islamic Azad University, Fars, Iran;; 2 Department of Biology, Shiraz Branch, Islamic Azad University, Shiraz, Iran

**Keywords:** Bone marrow, Mesenchymal stem Cells, Spermatogenesis, Infertility, Hamster

## Abstract

**BACKGROUND:**

Infertility is a serious social problem in advanced nations, with male factor in half of all cases of infertility. This study was conducted to determine the regenerative effect of bone marrow-derived stem cells in spermatogenesis of infertile hamster.

**METHODS:**

Twelve adult male hamsters were equally divided into azoospermic and control groups. Busulfan was intraperitoneally used for induction of azoospermia, while the right testis was treated with bone marrow-derived stem cells (10^6^ BM-SCs), labeled with sterile trypan blue, 35 days after busulfan injection. The left testis served as positive control for azoospermia. Sixty days after cell transplantation, the animals were euthanized and both testes were removed and evaluated histologically.

**RESULTS:**

BM-SCs were spindle-shaped, adherent to the culture flasks and had positive expression of CD29 and CD73 and negative expression of CD45. Alcian blue staining confirmed differentiation of BM-SCs into chondrocytes. Karyotyping denoted to stability of chromosomes. Treatment with busulfan in seminiferous tubules resulted into distruption of spermatogenesis. After two months in busulfan treatment group, seminiferous tubular atrophy and germinal epitheliums degenerations were noticed with no spermatozoa in epididymis. After treatment of busulfan group with BM-SCs, spermatogonia, primary spermatocytes, spermatids and sperms were present in seminiferous tubules.

**CONCLUSION:**

As cell transplantation in seminiferous tubules resulted into a rapid repair of pathological changes, BM-SCs can be recommended an effective treatment measure in azoospermia. It seems that more studies are necessary to confirm the use of this technique in treatment of azoospermia and infertility in human.

## INTRODUCTION

Infertility is a serious social problem in advanced nations, with male factor infertility accounting for approximately half of all cases of infertility.^1^ Men in reproductive age can deliver 96 million sperms per ejaculation.^2^ A complete absence of spermatozoa in the ejaculate without implying an underlying etiology, called azoospermia, affects approximately 1% of the male population and 10-15% of the males with infertility.^3^ In about 2/3 of these men, azoospermia is associated with a spectrum of untreatable testicular disorders that results in spermatogenic failure (SF), which has been recognized as the most severe presentation of male infertility.^4^

Azoospermia is classified as obstructive azoospermia (OA) or non-obstructive azoospermia (NOA), each having very different etiologies and treatments.^5^ OA comprises 40% of azoospermia cases and is typically accompanied by preservation of normal exocrine and endocrine functions and a normal spermatogenesis in the testis.^6^ OA is the consequence of physical blockage to the male excurrent ductal system and can happen in any region between the rete testis and the ejaculatory ducts.^7^

NOA affects approximately 60% of azoospermic males and includes non-obstructive causes of azoospermia, such as toxic exposures or abnormal testicular development.^8^ NOA results from either primary testicular failure (elevated LH, FSH, small testes affecting up to 10% of men presenting with infertility), secondary testicular failure (congenital hypgonadotropic hypogonadism with decreased LH and FSH, small testes), or incomplete or ambiguous testicular failure (either increased FSH and normal volume testes, normal FSH and small testes, or normal FSH and normal testis volume).^6^

Various treatments for male infertility exist including in vitro fertilization [particularly intracytoplasmic sperm injection (ICSI)] and microdissection testicular sperm extraction (MD-TESE) coupled with ICSI. However, there is currently no effective treatment for patients in whom there is an absence of mature sperm in the testes. Although evidence suggests that many patients with azoospermia have a genetic predisposition to the condition, the cause in most cases remains unknown.^9^

In spite of hormonal or surgical treatments, in recent years, more attention has been centralized on the cell and stem cell therapy. Mesenchymal stem cells have been isolated from different sources such as bone marrow,^10^ adipose tissue,^11^ endometrium,^12^ menstrual blood^13^ and dental pulp.^14^ Stem cell therapy has potential to develop more and being a choice for treatment of male infertility due to dysfunction of germ cells and their inability to differentiate or proliferate.^15^ There are some reports on transplantation of MSCs to induce spermatogenesis in azoospermic models.^16^ Therefore, this study was undertaken to use busulfan for induction of azoospermia and BM-MSCs for its cell therapy and further spermatogenesis in a hamster model.

## MATERIALS AND METHODS

Twelve adult male albino hamsters (95±5 g) were provided from Laboratory Animal Center, Shiraz University of Medical Sciences, Shiraz, Iran and kept in polypropylene cage at a controlled temperature (20-22°C) under a period of 12h lightness/darkness (7.00-19.00 lightning) with free access to food and water. All animal procedures were approved by the Ethical Committee of our institution. The hamsters were divided into two groups of azoospermic and control (n=6) animals. The control group was applied as cell donor (n=3) and also the left testis (n=3) was used as negative control. In the azoospermic group, the right testis was treated with bone marrow-derived stem cells (BM-SCs) and the left testis was considered as positive control.

BM-SCs were isolated from femur. Both ends of the femoral bone were cut and the bone marrow (BM) was flushed out using a 10 ml syringe filled with Dulbecco’s modified eagle medium (DMEM; Biovet, Bulgaria) and 1% penicillin streptomycin (Sigma, USA). Then, BM was transferred on ice to the laboratory, under sterile conditions and BM-SCs culture was performed. 

Briefly, bone marrow was diluted with an equal volume of DMEM and centrifuged at 1200 rpm for 7 min. The precipitate was plated in 75 cm^2^ flasks containing DMEM supplemented with 10% fetal bovine serum (FBS; Biovet, Bulgaria), 1% penicillin and streptomycin and 1% l-glutamine (Sigma, USA) and transferred into CO_2_ incubator at 37°C with 5% CO_2_ and saturated humidity. The medium was changed every 72 h. Adherent cells were subcultured when they were 80% confluent by washing twice with phosphate buffer saline (PBS, Gibco, USA) and using 0.25% trypsin (Gibco, USA) for 3-4 min. To inactivate enzyme activity, the same volume of supplemented DMEM media was added. Cell passage was continued until passage 4.

To coordinate between azoospermia model preparation and cell isolation and characterization, it was necessary to cryopreserve the isolated cells for further steps of the research. For this purpose, the confluent flasks of BM-SCs in passage 4 were treated with 0.25% trypsin (Gibco, USA) for 3-4 min and then the enzyme was inactivated by equal amount of supplemented DMEM media. The cell suspension was centrifuged at 1500 rpm for 5 min and the supernatant was removed and the precipitate was suspended in mixture of 50% DMEM media, 40% FBS, and 10% dimethyl sulfoxide (DMSO; MP Bio) at a density of 2×10^6^ viable cells/ml and was aliquoted into sterile plastic labeled cryovials. They were frozen in -20°C for one hour and then in -70°C for 24 h, and finally transferred to liquid nitrogen for long-term storage. 

Whenever BM-SCs were needed, they were taken out for thawing and culturing. To thaw the cells, the cryovials were removed from the liquid nitrogen and placed in a 37°C water bath. Then, cell culture DMEM medium was added and the mixture was centrifuged at 1500 rpm for 5 min. The precipitated cells were plated into a culture flask and transferred into an incubator with 5% CO_2_ and saturated humidity at 37°C. The cells were subcultured once after thawing.

Busulfan was used for induction of azoospermia. The animals were intraperitoneally injected by 40 mg/kg of busulfan (Busilvex®, Pierre Fabre Medicament, Boulogne, France) in 2 doses with 21 days interval to disrupt spermatogenesis. Thirty five days after the last busulfan injection, the animals were anesthetized using ketamine (40 mg/kg, Woerden, Netherlands) and xylazine (0.5 mg/kg Alfazyne®, 2%, Woerden, Netherlands). They were placed in dorsal recumbency and the abdominal area was prepared for further surgery. A 1 cm incision was performed at the abdominal midline to reach the peritoneal cavity. Under a Zeiss OPMI operating microscope (Carl Zeiss Meditec, Jena, Germany), the attached fat pad to the left seminiferous and testis was pulled gently by an iris forceps till the testis was taken out and was visible clearly. 

A pulled glass pipette was connected to the tube. For injection of BM-SCs in testis, they were labeled by addition of sterile trypan blue (1:1, v/v), served as a marker to monitor the success of the injection, loaded into the polyethylene tubing attached to a 1 ml syringe. The cell suspension was gently forced into the pipette by pressing the syringe. The seminiferous tubules were identified using stereomicroscope while 100 µl of BM-SCs’ mixture (10^6^ cells) were injected into the lumen of the seminiferous tubules of the busulfan-treated testis. The testis was returned to the abdominal cavity and the abdominal wall and skin were sutured. The right testis was considered as control.

About 1×10^4^ BM-SCs were transferred into two 35 mm culture dishes of control medium composed of DMEM, (BioWest, France) supplemented with 10% FBS (BioIdea, Iran), 1% penicillin/streptomycin (BioWest, France,) and 1% L-glutamine (BioIdea, Iran) and osteogenic medium consisted of DMEM-F12, 10% FBS, 1% penicillin-streptomycin, L-glutamine, 50 μg/ml L-ascorbic acid-2-phosphate (Sigma, USA), 10-7 M dexamethasone (Sigma, USA), and 10 mM β-glycerophosphate (Sigma, USA). The media was changed every 3 days and then, cells were fixed in 70% ethanol for 15 min and stained with 2% alizarin red S to assess the mineralization under light microscope.

Sixty days after cell transplantation, the animals were euthanized and both testes were removed and transferred into 10% formalin buffer solution. After fixation, they were embedded in paraffin, and histopathologic sections were made from each block. Five vertical sections from the polar and the equatorial regions with 5 µm thickness were hematoxylin-eosin stained and examined under light microscope for any spermatogenic activity.

All tubules were evaluated for the presence of any spermatogonia, spermatocytes, and spermatids. Ten identical circular transverse sections were performed in tubules, each in a different areas of the testes using a systematic random protocol to assess the stereological indices. The mean seminiferous tubule diameter (d) was determined by taking the average of two diameters, D_1_ and D_2_, at right angles. Cross-sectional area (A_c_) of the seminiferous tubules was determined using the equation of A_c_=πD^2^/4, where π is equal to 3.142 and D as the mean diameter of seminiferous tubules.

A testis was rated for its spermatogenic potential by a modified spermatogenic index on a scale of 0 to 6. The index was based on the appearance of the spermatogenic cells throughout the testis and included number of cell layers, types of cells, and the presence of late spermatids in the seminiferous tubules. The index and criteria were as follows: 0, no spermatogenic cells; 1, only spermatogonia present; 2, spermatogonia and spermatocytes present; 3, spermatogonia, spermatocytes and round (early) spermatids present with <50 late spermatids per tubule; 4, spermatogonia, spermatocytes, and round spermatids present; and up to 50-100 late spermatids per tubule; 5, spermatogonia, spermatocytes, and round spermatids present; and up to 100-150 late spermatids per tubule; and 6, all cell types present and >150 late spermatids per tubule.

The data were analyzed using SPSS software (version 18, Chicago, IL, USA). One way ANOVA, followed by LSD post-hoc tests were used for statistical analyses. The spermatogenesis index of seminiferous tubules was compared using Mann-Whitney U test. P<0.05 was considered significant.

## Results

**Fig. 1:**
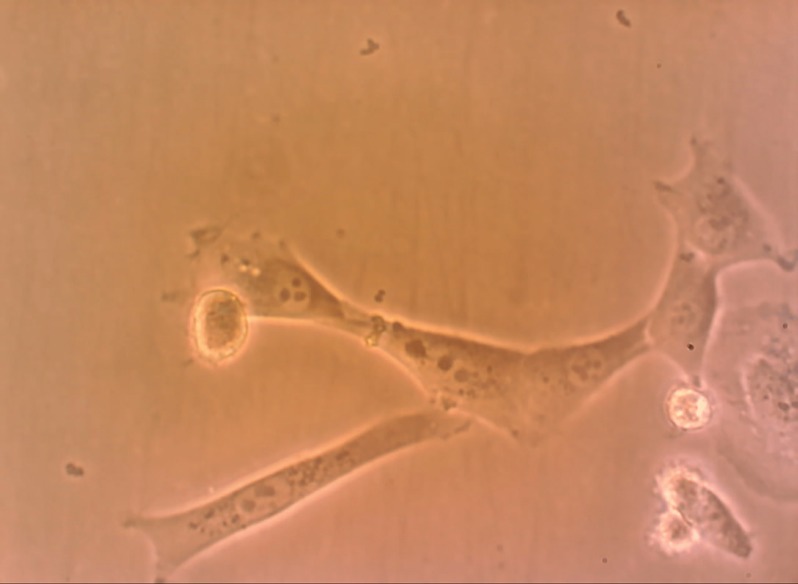
Fibroblast-like morphology of bone marrow derived stem cells in hamster.

**Fig. 2:**
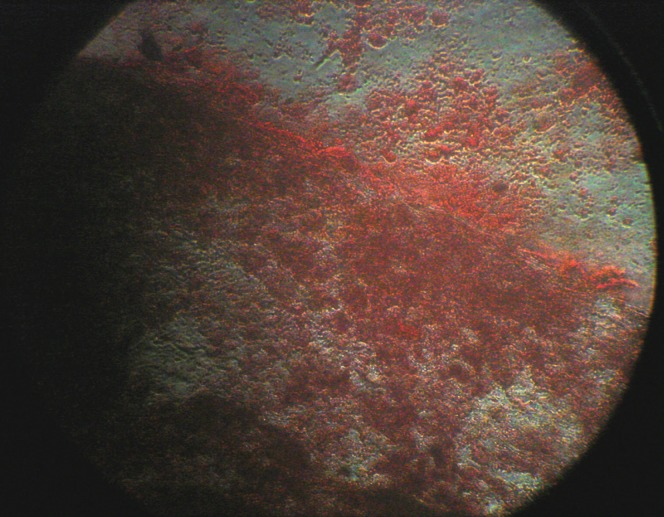
Positive alizarin red staining of bone marrow derived stem cells in presence of osteogenic medium after 21 days.

**Fig. 3:**
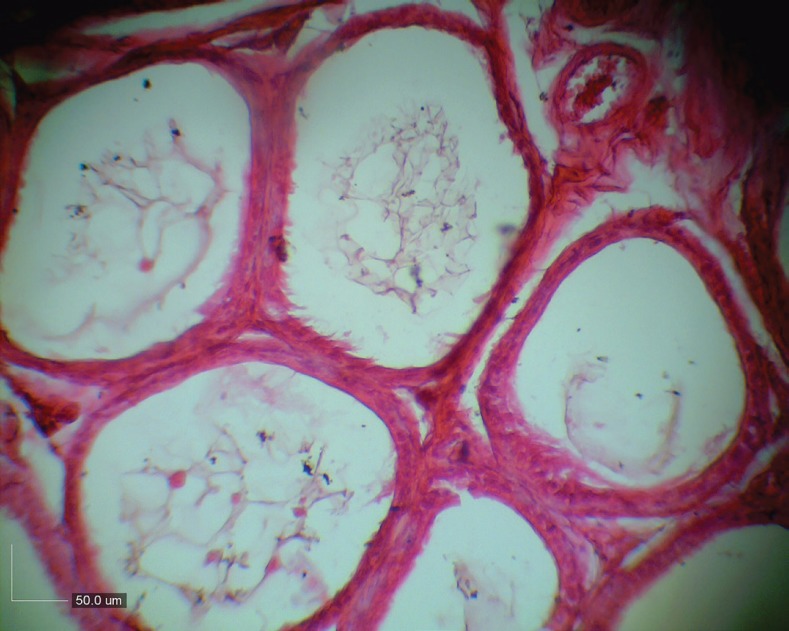
Treatment with busulfan, in seminiferous tubules resulted into distruption of spermatogenesis.

All BM-SCs up to passage 3 were spindle-shaped in morphology, adherent to the culture flasks (Figure 1). Analysis of alizarin red S staining showed that BM-SCs had osteogenic differentiation potential (Figure 2). Treatment with busulfan, in seminiferous tubules resulted into distruption of spermatogenesis (Figure 3). 

**Fig. 4:**
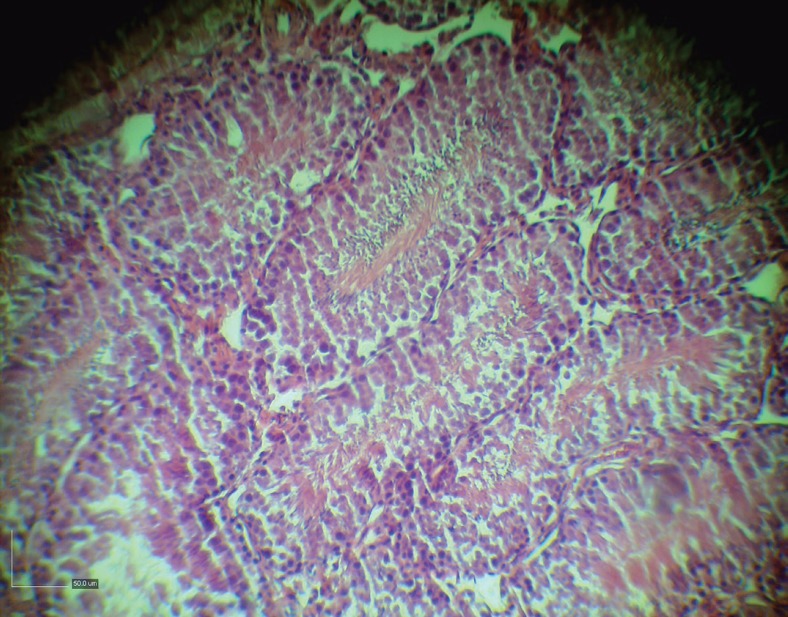
Presence of spermatogonia, primary spermatocytes, spermatids and sperms in the seminiferous tubules after treatment of busulfan treatment azoospermic group with BM-SCs.

**Fig. 5:**
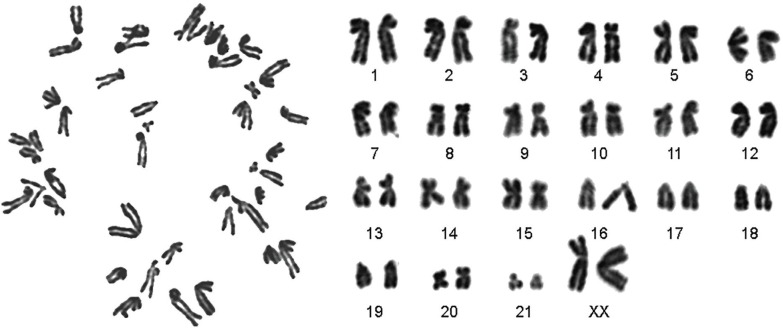
Karyotype of bone marrow derived stem cells in hamster.

After treatment of busulfan treatment azoospermic group with BM-SCs, spermatogonia, primary spermatocytes, spermatids and sperms were present in the seminiferous tubules (Figure 4). Figure 5 denotes to a normal karyotype of bone marrow derived stem cells in hamster and the stability in chromosomes.

## DISCUSSION

There are some reports on transplantation of MSCs to induce spermatogenesis in azoospermic models.^16^ Lassalle *et al.* (2008) injected BM-SCs into testis to induce differentiation of these cells into germ cells but, they did not notice any differentiation.^17^ In another study in mouse and human, adult BM-SCs grown in-vitro in the presence of retinoic acid were found to express germ cell markers, but there was still a failure to undergo spermatogenesis after transplantation into testes.^18^

Lue *et al.* (2007) showed that transplanting BM-SCs into testis of a busulfan-treated infertile mouse model could differentiate into germ, sertoli and leydig cells.^19^ Cakici *et al.* (2013) also demonstrated that treatment of busulfan-treated rat testes with adipose tissue derived MSCs resulted into morphologically normal spermatogenesis in some tubules after 12 weeks.^20^ Monsefi *et al.* (2013) showed that transplanted BM-SCs could differentiate into germinal cells in seminiferous tubules of Wistar rats.^21^

Our study showed that injected BM-SCs could induce spermatogenesis. As repair in damaged testis is one of the most limitations in infertility treatment and azoospermia,^22^ BM-SCs as hypo-immunogenic cells with immunosurveillance or immunosuppression properties, so they can be a suitable choice for allogeneic cell transplantation.^23^ Immunomodulatory effects of BM-SCs on antisperm antibody production in allogeneic settings in mice after testis rupture were previously shown.^24^ As Sertoli cells are immune tolerant cells,^25^ they provide the survival of the donor cells after transplantation for any immune response. 

We showed that spermatogenesis index in seminiferous tubules after cell transplantation was identical to the control group. Seminiferous tubules are responsible for cyclic regulation of spermatogenesis, and Sertoli cells provide the microenvironment for spermatogonial proliferation and differentiation,^25^ so BM-SCs in our study resulted into reconstitution of the tubular microenvironment and proliferation of inactivated germinal cells in the host tubules.

It was previously demonstrated that transplanted mouse BM-SCs can form germ cells in-vivo.^19^ Also, BM-SCs was shown to differentiate into germ cells and spermatozoa in-vitro.^26^ Nayernia et al.^18^ demonstrated that BM-SCs in mice can differentiate into early germ cells in-vitro and in-

Allogeneic transdifferentiation of BM-SCs into spermatogenic-like-cells and the fertility recovery in rat after injection of busulfan into seminiferous tubules of recipient^27^ and in cases of testicular torsion^28^ azoospermia. Cakici *et al.*^20^ demonstrated that adipose-tissue-derived MSCs can recover fertility after injection of busulfan in rat. Chen et al.^29^ showed that transplantation of human umbilical cord MSCs into seminiferous tubules of immunodeficient mouse can lead to sperm differentiation that denotes to efficacy of cell transplantation in azoospermia.

Current medical therapies in azoospermia are hormonal or surgical methods with little benefits in non-obstructive azoospermia.^30^ Busulfan as a chemotherapeutic agent can affect cells with high division activities such as germ cells and destroy spermatogonial stem cells,^31^ and inhibits the next mitosis in cells in the G1 phase, but it has no effect on DNA synthesis.^31^

vivo that confirm our findings.

Three mechanism might be responsible to recover testicular function during the tissue regeneration process by MSCs: (i) They may differentiate into the target cells via appropriate induction conditions;^32^ (ii) These cells secrete growth factors to stimulate the resident stem cells to restore the host cell function;^33^ and (iii) MSCs merged with the endogenous cells recover the injured tissue function.^34^

As cell transplantation in seminiferous tubules of hamster resulted into a rapid repair of pathological changes in testicular tubules, BM-SCs can be recommended as an effective treatment measure in cases of azoospermia. It seems that more studies are necessary to confirm the use of this technique in treatment of azoospermia and infertility in human.
